# Neoadjuvant radiation followed by resection versus upfront resection for locally advanced pancreatic cancer patients: a propensity score matched analysis

**DOI:** 10.18632/oncotarget.18091

**Published:** 2017-05-23

**Authors:** Xing Chen, Geng Liu, Kaiqiang Wang, Guodong Chen, Jinjin Sun

**Affiliations:** ^1^ Department of Hepatopancreatobiliary Surgery, The Second Hospital of Tianjin Medical University, Tianjin, China

**Keywords:** LAPC, neoadjuvant radiation, resection, CSS, SEER

## Abstract

**Background and aim:**

To compare cancer-specific survival (CSS) between patients who received neoadjuvant radiation followed by resection (NRR) and those who received upfront resection (UR) for locally advanced pancreatic cancer (LAPC).

**Methods:**

A total of 772 LAPC patients who underwent curative-intent surgical resection with or without neoadjuvant radiation from 2004 to 2013 were identified from the Surveillance, Epidemiology, and End Result (SEER) database. Propensity score matching (PSM) was conducted to eliminate possible bias. Kaplan-Meier method was used to analyze long-term outcome. Independent risk factors of CSS were predicted by Cox proportional hazards model. Subgroup analyses were done according to 5 variables.

**Results:**

The propensity score model matched 196 patients from the whole cohort. Neoadjuvant radiation was an independent predictor of CSS no matter before or after PSM. After PSM, the 1-, 3-, 5-year CSS rates of NRR group were 82.7%, 39.2% and 17.1%, while 64.3%, 19.9% and 12.4% for UR group. The median CSS for NRR group was 25 months, while 17 months for UR group. In subgroup analyses, CSS rates or median CSS of NRR group were still superior to those of UR group in married, unmarried, pancreatic adenocarcinoma, G1+G2, G3+G4, N0 stage, N1 stage and M0 stage subgroups, but no differences were found in other histological types and M1 stage subgroups. Other predictors of CSS included histological type, tumor grade and marital status.

**Conclusions:**

Neoadjuvant radiation followed by resection has a significant survival benefit compared with upfront resection in LAPC patients. Therapeutic strategy for LAPC patients should be further explored.

## INTRODUCTION

According to the latest statistics, cancer of pancreas was the fourth cause of death among all types of cancer, and 45090 patients were estimated to die of this malignancy in 2017 in the USA [1]. Pancreatic carcinoma was characterized by a dismal prognosis, with five-year relative survival rate of merely 8% [1]. One cause of poor survival was due to lack of specific signs and symptoms, and a considerable part of patients presented with advanced or metastatic disease. 52% patients were reported to have distant metastasis at diagnosis [1]. Treatment strategies for early resectable and metastatic disease were relatively clear and definite, namely resection plus sequential adjuvant chemotherapy for the former and systemic chemotherapy or best supportive care for the latter [2]. However, that was not the case for locally advanced patients (T4, with celiac trunk or superior mesenteric artery involved). Although systemic chemotherapy and support care were recommended for those patients [2], surgeons never stopped trying surgical resection. After all, surgical resection remained the only hope for complete cure. Nevertheless, results were inconsistent. Some researchers found that pancreatectomy plus artery resection yielded poor long-term survival, with median overall survival (OS) ranging from 8.5-11.5 months [3–7]. By contrast, investigations from some studies were much more encouraging, with median OS ranging from 18-35 months [8, 9]. The difference in survival could be, in part, attributed to neoadjuvant therapy besides heterogeneity of study subjects, as high proportion of patients in the latter studies received various neoadjuvant regimens.

Therefore, to further investigate relationship between neoadjuvant therapy and pancreatic cancer patient prognosis, as well as potential underlying mechanisms, we use data from the Surveillance, Epidemiology, and End Result (SEER) database to explore the impact of neoadjuvant radiation on cancer-specific survival (CSS) in LAPC patients who received surgical resection with or without preoperative neoadjuvant radiation. Hopefully, those results will guide therapeutic decision-making in the future.

## RESULTS

### Propensity score matching (PSM)

A total of 772 locally advanced pancreatic cancer patients treated by curative-intent surgery with or without neoadjuvant radiation were identified from the 2004-2013 SEER database, and they were divided into two groups according to treatment strategy: UR (upfront resection) and NRR (neoadjuvant radiation followed by resection) group. Before matching, there were 670 patients in the former group and 102 in the latter. After 1:1 matching, there were 98 patients in each group respectively. The mean propensity score for UR and NRR group was 0.118±0.096 and 0.224 ± 0.128 before matching, and the mean propensity score for UR and NRR group was 0.212±0.119 and 0.213 ± 0.119 after matching. Distribution of covariates was adequately balanced in the matched data set, as shown in Table 1.

**Table 1:** Characteristics of UR and NRR group before and after propensity score matching (PSM)

Variable		Before PSM			After PSM		
		UR(n=670)	NRR(n=102)	P Value	UR(n=98)	NRR(n=98)	P Value
Age (y)	<60	245(36.6)	49(48.0)	0.026	45(45.9)	45(45.9)	1.000
	≥60	425(63.4)	53(52.0)		53(54.1)	53(54.1)	
Sex	Male	360(53.7)	53(52.0)	0.738	52(53.1)	52(53.1)	1.000
	Female	310(46.3)	49(48.0)		46(46.9)	46(16.9)	
Marital status	Married	434(64.8)	85(83.3)	0.001	82(83.7)	82(83.7)	1.000
	Unmarried	218(32.5)	16(15.7)		16(16.3)	16(16.3)	
	Missing	18(2.7)	1(1.0)		a	a	
Race	White	524(78.2)	85(83.3)	0.478	83(84.7)	81(82.7)	0.926
	Black	80(11.9)	10(9.8)		9(9.2)	10(10.2)	
	Others	66(9.9)	7(6.9)		6(6.1)	7(7.1)	
Insurance	Insured	393(58.7)	78(76.5)	0.002	82(83.7)	86(87.8)	0.414
	Uninsured	61(9.1)	4(3.9)		16(16.3)	12(12.2)	
	Missing	216(32.2)	20(19.6)		a	a	
Site	Pancreas head	393(58.7)	59(57.9)	0.926	70(71.4)	70(71.4)	1.000
	Pancreas body and tail	169(25.2)	25(24.5)		28(28.6)	28(28.6)	
	Missing	108(16.1)	18(17.6)		a	a	
Surgery	Pancreatectomy	136(20.3)	18(17.7)	0.064	17(17.3)	19(19.4)	0.712
	PD	472(70.4)	81(79.4)		81(82.7)	79(80.6)	
	Missing	62(9.3)	3(2.9)		a	a	
LR	Yes	545(81.4)	91(89.3)	0.061	88(89.8)	89(90.8)	0.809
	No	112(16.7)	8(7.8)		10(10.2)	9(9.2)	
	Missing	13(1.9)	3(2.9)		a	a	
Size (cm)	≤4	332(49.6)	55(54.0)	0.440	60(61.2)	56(57.1)	0.561
	>4	258(38.5)	39(38.2)		38(38.8)	42(42.9)	
	Missing	80(11.9)	8(7.8)		a	a	
Histological type	PA	548(81.8)	95(93.1)	0.004	91(92.9)	91(92.9)	1.000
	Others	122(18.2)	7(6.9)		7(7.1)	7(7.1)	
Grade	G1+G2	318(47.5)	39(38.3)	0.000	56(57.1)	57(58.2)	0.885
	G3+G4	209(31.2)	23(22.5)		42(42.9)	41(41.8)	
	Missing	143(21.3)	40(39.2)		a	a	
N stage	N0	234(35.0)	57(55.9)	0.000	54(55.1)	53(54.1)	0.886
	N1	411(61.3)	45(44.1)		44(44.9)	45(45.9)	
	Missing	25(3.7)	0(0.0)		a	a	
M stage	M0	498(74.3)	95(93.1)	0.000	90(91.8)	91(92.9)	0.778
	M1	160(23.9)	7(6.9)		8(8.2)	7(7.1)	
	Missing	12(1.8)	0(0.0)		a	a	

^a^Missing cases were distributed into two groups after multiple imputation.

^b^n (%) for categorical variables.

UR: upfront resection; NRR: neoadjuvant radiation followed by resection; PD: pancreaticoduodenectomy; LR: lymph nodes resection; PA: pancreatic adenocarcinoma.

### Predictors of cancer-specific survival (CSS)

Neoadjuvant radiation, histological type, tumor grade and marital status were proved to be independent predictors of CSS in the unmatched data set on multivariable analysis (Table 2). After matching, neoadjuvant radiation, histological type and tumor grade were still independently correlated with CSS on multivariable analysis, leaving marital status behind (Table 2). What was noteworthy was that neoadjuvant radiation was an independent predictor of CSS no matter before (HR: 2.315, 95% CI: 1.399, 3.831, P=0.001) or after (HR: 1.866, 95% CI: 1.294, 2.688, P=0.001) PSM.

**Table 2:** Univariate and multivariate analysis of prognostic factors of cancer-specific survival before and after propensity score matching (PSM)

Variable	Before PSM				After PSM			
	Univariate analysis	Multivariate analysis			Univariate analysis	Multivariate analysis		
	P Value	HR	95% CI	P value	P Value	HR	95% CI	P value
Age (y), <60/≥60	0.004				0.880			
Sex (male/female)	0.471				0.145			
Marital status (married/unmarried)	0.015	1.425	(1.057,1.919)	0.02	0.763			
Race (white/black/others)	0.329				0.161			
Insurance (insured/uninsured)	0.710				0.072			
Neoadjuvant radiation (yes/no)	0.000	2.315	(1.399,3.831)	0.001	0.003	1.866	(1.294,2.688)	0.001
Site (pancreas head/body and tail)	0.737				0.861			
Surgery (pancreatectomy/PD)	0.106				0.826			
LR (yes/no)	0.036				0.365			
Size (≤4cm/>4cm)	0.039				0.707			
Histological type (PA/others)	0.000	2.564	1.642,4.000)	0.000	0.016	3.125	(1.355,7.194)	0.008
Grade (G1+G2/G3+G4)	0.000	1.621	(1.216,2.161)	0.001	0.008	1.729	(1.203,2.486)	0.003
N stage (N0/N1)	0.164				0.616			
M stage (M0/M1)	0.011				0.426			

UR: upfront resection; NRR: neoadjuvant radiation followed by resection; PD: pancreaticoduodenectomy; LR: lymph nodes resection; PA: pancreatic adenocarcinoma.

### NRR group versus UR group

**Figure 1:**
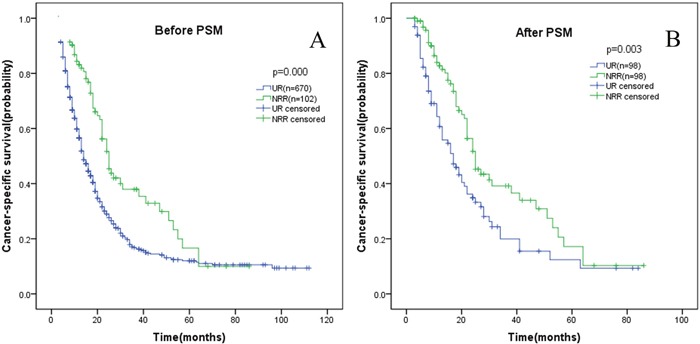
Kaplan-Meier curve of cancer-specific survival comparing UR group with NRR group before **(A)** and after **(B)** propensity score matching (PSM). UR: upfront resection; NRR: neoadjuvant radiation followed by resection.

**Figure 2:**
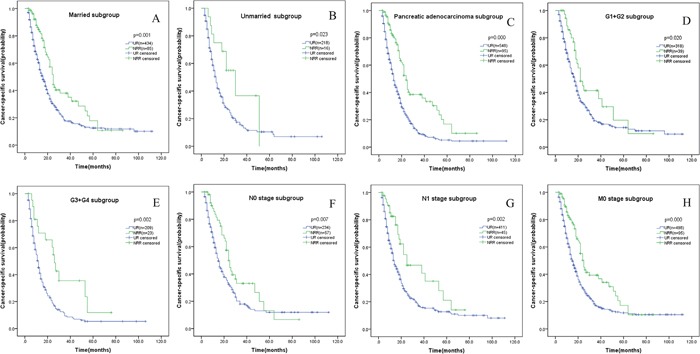
Kaplan-Meier curve of cancer-specific survival comparing UR group with NRR group in different subgroups **(A)** Married subgroup; **(B)** unmarried subgroup; **(C)** pancreatic adenocarcinoma subgroup; **(D)** G1+G2 subgroup; **(E)** G3+G4 subgroup; **(F)** N0 stage subgroup; **(G)** N1 stage subgroup; **(H)** M0 stage subgroup. UR: upfront resection; NRR: neoadjuvant radiation followed by resection.

Before PSM, significant differences were observed in terms of age, marital status, insurance, histological type, tumor grade, N stage, M stage between the two groups. In comparison to UR group, more patients in NRR group were less than 60 years old, married and insured; more patients were pathologically confirmed pancreatic adenocarcinoma; less patients were histologically validated to have G3+G4 tumor grade disease; less patients were proved to have regional lymph nodes or distant metastasis disease (Table 1). Before PSM, the 1-, 3-, 5-year CSS rates of NRR group were 83.2%, 37.9% and 16.6%, which were significantly superior to those of UR group, namely 56.6%, 16.8% and 12.0%. The median CSS for NRR group was 25 months, while 14 months for UR group (Table 3, Figure 1). After PSM, 1-, 3-, 5-year CSS rates of NRR group were 82.7%, 39.2% and 17.1%, which were still significantly higher than those of UR group, namely 64.3%, 19.9% and 12.4%. The median CSS for NRR group was 25 months, while 17 months for UR group (Table 3, Figure 1). In subgroup analyses, CSS rates or median CSS of NRR group still had advantages over those of UR group in most subgroups, including married, unmarried, pancreatic adenocarcinoma, G1+G2, G3+G4, N0 stage, N1 stage and M0 stage subgroups (Figure 2), while no differences were found in other histological types and M1 stage subgroups (Figures were not shown).

**Table 3:** Cancer-specific survival (CSS) rate and median CSS (months) before and after propensity score matching (PSM)

Group	Before PSM				After PSM			
	Cancer-specific survival rate			median CSS	Cancer-specific survival rate			median CSS
	1-year	3-year	5-year		1-year	3-year	5-year	
The whole cohort	60%	19.4%	12.7%	16	71.6%	29.4%	14.8%	22
NRR group	83.2%	37.9%	16.6%	25	82.7%	39.2%	17.1%	25
UR group	56.6%	16.8%	12.0%	14	64.3%	19.9%	12.4%	17

## DISCUSSION

Vascular invasion (especially arterial invasion) in LAPC patients meant great operation complexity and aggressive tumor biological behavior, with the prior one gradually solved by accumulating experience and continuously improving surgical devices. Lower perioperative morbidity and mortality rates and longer survival were reported in recent studies than in earlier ones [8–14]. For the latter one, adjuvant and neoadjuvant therapy was inevitable. Neoadjuvant therapy held one prominent advantage over adjuvant therapy, as some patients receiving surgical treatment first were not able to finish planned adjuvant treatment because of postoperative complications caused by aggressive procedures or early recurrence. 26% to 38% of patients were reported not to receive postoperative chemotherapy by prospective observation from large-volume academic centers [15, 16]. Although high-level evidence was not available, the latest NCCN guidelines recommended neoadjuvant therapy in borderline resectable pancreatic adenocarcinoma patients [2]. However, less was known about neoadjuvant therapy in LAPC patients, in which vascular invasion was more severe. By contrast, survival benefit of neoadjuvant therapy has been investigated in various advanced or locally advanced cancers of other sites, including: lung cancer, gastric cancer, bladder cancer, and prostate cancer [17–20].

The present SEER data demonstrated that neoadjuvant radiation with sequential resection prolonged survival duration compared with upfront resection for LAPC patients (median CSS: 25 months vs 14 months, P=0.000). Similar results were found in a multicenter prospective observational cohort, in which median disease-free survival and OS of patients amenable to surgery after FOLFIRINOX and chemoradiation induction therapy were 22.5 and 24.9 months respectively. Besides, authors reported that 36.3% initially unresectable patients (n=28) underwent resection after neoadjuvant therapy, and R0 resection rate was 89% [21]. A Memorial Sloan Kettering Cancer Center trial turned out that nearly one third of initially unresectable patients (n=31) underwent resection after FOLFIRINOX and chemoradiation induction therapy, and R0 resection rate was 55%. Unfortunately, median OS was not reported in this study as it was not reached yet [22]. A recent trial comparing 3 different neoadjuvant regimens in LAPC patients observed the highest resection rate in FOLFIRINOX group (76/125 patients, 60.8%). Median OS was 22 months in this study [23]. Another retrospective study of neoadjuvant therapy in LAPC patients allowed 10 of 51 patients (20%) to be successfully R0 resected and a satisfactory 3-year OS rate of 67% [24]. It was easy to conclude that these studies were relatively small-sized ones mainly focusing on resectability change before and after neoadjuvant therapy. In comparison, the current study provided with a direct comparison between surgery following neoadjuvant radiation and upfront surgery to highlight the role of neoadjuvant radiation based on a large-sized sample. Besides, neoadjuvant radiation, histological type, tumor grade and marital status were found to be significantly correlated with CSS in our study. Notably, lymph node metastasis was not an independent predictor in our study. We guessed that the effect of lymph node metastasis was covered up by neoadjuvant radiation, as neoadjuvant radiation may eliminate some lymph node metastasis, but it could not change histological type and tumor grade.

Opponents of neoadjuvant therapy raised 2 major concerns against its use, namely increased operative mortality and morbidity rates and the possibility of disease progression to be unresectable during the course of neoadjuvant therapy [25]. However, a project study of 992 pancreatic cancer patients by the Japanese Society of Hepato-Biliary-Pancreatic Surgery demonstrated that neoadjuvant therapy did not increase perioperative mortality and morbidity rates [26]. Similar results were confirmed by other researches [27–29]. Meanwhile, it was believed that most patients receiving neoadjuvant therapy would not progress to be unresectable by several studies [26, 29]. Progression leading to utter unresectability occurred in 3.2% and 7.4% patients in resectable group (6/185) and borderline resectable group (15/203) respectively after neoadjuvant therapy according to the above-mentioned Japanese project study, which was acceptable [26]. Reasonable inference could be made that those patients spared by neoadjuvant treatment would not survive long even if they underwent surgery first due to aggressive nature, and this kind of spare was one of the goals neoadjuvant therapy was designed for.

The main aim of neoadjuvant protocols was to downstage relatively advanced tumor to achieve a margin-negative resection and then a possibility of long-term survival [30]. Nevertheless, no difference was observed with regards to tumor size in two groups, indicating that tumor bulk shrinking was not the way neoadjuvant radiation improved survival. This finding was supported by other studies [30–32]. However, some studies held opposite opinions [33, 34]. The latest NCCN guidelines tended to recommend attempted surgery even no radiographic response after neoadjuvant therapy as long as no evident extrapancreatic progressive disease [2]. By contrast, the most significant differences observed between NRR and UR groups lied in N stage and M stage. N1 (regional lymph nodes metastasis) and M1 (distant metastasis, mainly distant lymph nodes metastasis) occurred in 44.1% and 6.9% patients in NRR group, significantly lower than those in UR group (61.3% and 23.9%). M1 represented distant lymph nodes and organ metastasis according to AJCC Staging Manual, but what was noteworthy was that M1 here indicated mainly distant lymph nodes metastasis as all cases included were subject to aggressive procedures such as pancreaticoduodenectomy. Tsutomu Fujii and Cristina R. Ferrone et al. also noted significantly low fraction of positive lymph nodes in neoadjuvant setting for locally advanced or borderline pancreatic cancer [29, 33]. Another commonly recognized prognostic factor was resection margin status, and R0 resection with adequate lymphadenectomy was the only chance for long survival. In studies simultaneously involving neoadjuvant therapy and immediate surgery, researchers identified significantly higher rate of R0 resection in pancreatic cancer patients of various stages receiving neoadjuvant therapy [27–29, 35]. Remarkably high R0 resection rate was reported in selective studies involving borderline resectable and locally advanced pancreatic carcinoma patients amendable to surgery after neoadjuvant therapy: ranging from 75.5% to 100% [21, 36–40]. In addition, perineural and lymphatic invasion rates were also reportedly significantly lower in neoadjuvant setting [29, 33]. By combination of the present SEER data and other researchers’ experience, it could be reasonably deduced that neoadjuvant therapy played its role mainly via eliminating undetected micrometastases around tumor bulk and in draining lymph nodes.

Admittedly, there were some limitations in our study. Firstly, it was an observational study vulnerable to confounding bias. Thus, we performed a 1:1 propensity score matched analysis to simulate a realistic scenario of two homogeneous populations receiving different treatment strategies. No matter before or after PSM, neoadjuvant radiation was confirmed to be a predictor for LAPC patients undergoing surgical treatment. Moreover, subgroup analyses were conducted according to confirmed risk factors in our study or others’. Survival duration still remained significantly longer in NRR group than in UR group in most subgroups, except for M1 stage and other histological types subgroups, and maybe because patients with or without neoadjuvant radiation in those two subgroups differed greatly. Secondly, surgical margin status and R0 resection rate were not available as the SEER database did not collect related data. Thirdly, the SEER database provided no information on administration of chemotherapy which were likely to be included as neoadjuvant regime. Therefore, our results should be interpreted with caution.

In summary, neoadjuvant radiation followed by resection demonstrated significant survival benefit in locally advanced pancreatic cancer patients, which still required confirmation from further phase III prospective randomized controlled trials. Our data provided encouragement and support for LAPC patients to participate in clinical trials evaluating the role of neoadjuvant therapy before planned curative-intent surgical resection.

## MATERIALS AND METHODS

### Data collection and patient selection

**Figure 3:**
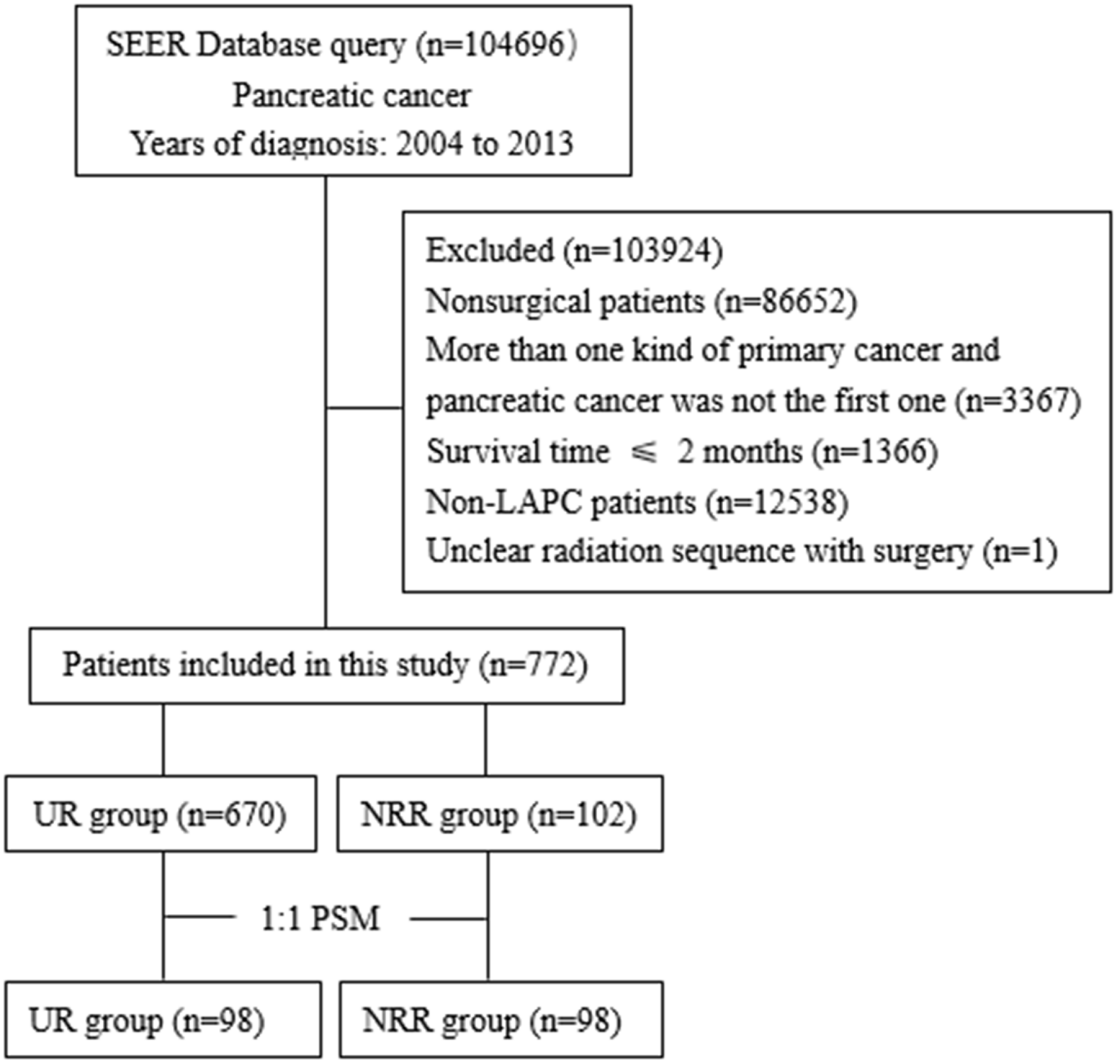
Flow chart of patient selection SEER: Surveillance, Epidemiology, and End Result database; LAPC: locally advanced pancreatic cancer; UR: upfront resection; NRR: neoadjuvant radiation followed by resection.

This study was conducted using public data from the SEER database, a population-based database covering approximately 28 percent of the US population, and demographic, clinical, operative and follow-up information needed was extracted using SEER*Stat Software Version 8.3.2 with permitted SEER ID 14005-Nov2015. Given the low administration rate of neoadjuvant therapy among patients of early years and progression in surgical technique and device, we only included patients diagnosed in the latest 10 years, namely from 2004 to 2013. “Site and morphology site recode ICD-0-3/WHO 2008=pancreas” was used to identify pancreatic cancer patients. All patients were histologically confirmed to have malignant disease of pancreas using International Classification of Disease for Oncology (ICD-O-3) codes such as 8140(adenocarcinoma), 8500(infiltrating duct carcinoma + noninfiltrating intraductal carcinoma). The inclusion criteria were as follows: (a) patients receiving surgical resection; (b) with one primary cancer only or with pancreatic cancer as the first one if there were more than one kind of cancer; (c) survival time more than 2 months; (d) locally advanced patients (T4, according to AJCC Cancer Staging Manual, Seventh or Sixth Edition); (e) definite radiation sequence with surgery. Selection process was shown in Figure 3.

### Definitions

“Unmarried” meant: single (never married), unmarried or domestic partner, divorced, and widowed; “uninsured” included uninsured and any medicaid, implying low income population; “pancreatic adenocarcinoma (PA)” indicated the most common four histological types, namely adenocarcinoma, infiltrating duct carcinoma, noninfiltrating intraductal carcinoma, and mucinous adenocarcinoma, while “others” implied less common histological types such as squamous carcinoma or adenosquamous carcinoma; local excision of tumor, partial pancreatectomy, and total pancreatectomy were brought into “pancreatectomy” group, while local or partial pancreatectomy and duodenectomy without or with subtotal gastrectomy, total pancreatectomy and subtotal gastrectomy or duodenectomy, and extended pancreatoduodenectomy were taken into “pancreatoduodenectomy (PD)” group.

### Statistical analyses

The primary endpoint of this study was cancer-specific survival, which was calculated from the date of diagnosis to the date of cancer-specific death. Deaths caused by pancreatic cancer were considered as events, while deaths attributed to other causes were considered as censored observations. For cancer-specific survival, univariate analysis was conducted using Kaplan-Meier method and compared using the log-rank test, while multivariate analysis was carried out using the Cox proportional hazards model. To compare differences of covariates between UR group and NRR group, *χ*^2^ or Fisher exact test (2-tailed) was adopted. To overcome confounding and selection biases derived from unbalanced perioperative factors among patients between the two groups, propensity score matching was conducted. To that end, multiple imputation was performed to fill in missing data in the first place, and then propensity score was calculated by logistic regression model [41]. The following variables age, sex, marital status, insurance status, histological type, tumor grade, N stage, M stage, tumor site, surgery, lymph nodes resection and tumor size were entered into the propensity model with caliper width set as 0.1 to produce a 1:1 nearest-neighbor matching between the two groups. For all tests, P<0.05 was considered statistically significant. All statistical analyses in this study were performed using software package SPSS 22.0 (SPSS Inc., Chicago) and Propensity Score Matching for SPSS, version 3.0.4 (Felix Thoemmes, Cornell University/University of Tübingen).

## Abbreviations

CSS: cancer-specific survival
NRR: neoadjuvant radiation followed by resection
UR: upfront resection
LAPC: locally advanced pancreatic cancer
SEER: Surveillance, Epidemiology, and End Result
PSM: propensity score matching
OS: overall survival
LR: lymph nodes resection
NCCN: National Comprehensive Cancer Network
PD: pancreaticoduodenectomy
ICD-O-3: International Classification of Disease for Oncology 3
AJCC: American Joint Committee on Cancer
PA: pancreatic adenocarcinoma.
